# The association between ondansetron use and mortality risk of traumatic brain injury patients: a population-based study

**DOI:** 10.3389/fphar.2024.1362309

**Published:** 2024-05-02

**Authors:** Ruoran Wang, Jing Zhang, Jianguo Xu, Min He

**Affiliations:** ^1^ Department of Neurosurgery, West China Hospital, Sichuan University, Chengdu, Sichuan, China; ^2^ Department of Critical Care Medicine, West China Hospital, Sichuan University, Chengdu, Sichuan, China

**Keywords:** traumatic brain injury, ondansetron, 5-HT3 receptor antagonist, mortality, population study

## Abstract

**Background:**

Traumatic brain injury (TBI) patients suffer high risks of mortality. Ondansetron has been verified to be effective in improving the prognosis of some kinds of critically ill patients. We design this study to explore whether ondansetron use is associated with lower risks of mortality among TBI patients.

**Methods:**

TBI patients from the Medical Information Mart for Intensive Care-III were collected. The usage of ondansetron, including intravenous injection and oral tablet, since admission to the Beth Israel Deaconess Medical Center between 2001 and 2012 was identified. Univariate and multivariate logistic regression were performed to analyze the relationship between the ondansetron use and mortality of TBI patients. Propensity score matching (PSM) was utilized to generate balanced cohorts of the non-ondansetron use group and ondansetron use group. Sub-group analysis was performed to verify the association between the ondansetron use and mortality of TBI patients in different TBI severity levels after PSM.

**Results:**

In TBI cohorts before PSM, the usage incidence of ondansetron was 37.2%. The 30-day mortality was significantly lower in the ondansetron group (*p* < 0.001). The multivariate logistic regression showed that ondansetron was associated with the lower mortality of TBI patients (*p* = 0.008). In TBI cohorts after PSM, the 30-day mortality of the ondansetron group was lower than that of the non-ondansetron group, although without statistical significance (*p* = 0.079). Logistic regression indicated ondansetron use was significantly associated with the lower mortality of moderate-to-severe TBI (*p* < 0.001) but not mild TBI (*p* = 0.051). In addition, Cox regression also presented that ondansetron use was significantly associated with the lower mortality of moderate-to-severe TBI (*p* < 0.001) but not mild TBI (*p* = 0.052).

**Conclusion:**

Ondansetron usage is associated with a lower mortality risk of moderate-to-severe TBI but not mild TBI patients. Ondansetron may be a novel adjunctive therapeutic strategy to improve the prognosis of moderate-to-severe TBI patients.

## 1 Introduction

The incidence of traumatic brain injury (TBI) is estimated to be 69 million per year around the world (Dewan et al., 2018). Nearly 44% of moderate-to-severe TBI survivors suffer from long-term disability and have a subsequent mortality rate of 16.5% per year (Brooks et al., 2013; Pozzato et al., 2019). Many novel therapies have been explored and developed for TBI, such as hyperbaric oxygen, deep brain stimulation, and erythropoietin. An important component of works improving the prognosis of TBI is developing novel drugs for neuroprotection, such as statin, glibenclamide, and inosine.

As a serotonin 5-hydroxytryptamine (5-HT3) receptor antagonist, ondansetron is commonly used for preventing and alleviating nausea and vomiting in patients receiving radio-chemotherapy or undergoing surgery. In addition to decreasing vomit-related complications, ondansetron has also been verified to improve the prognosis of critically ill patients, including those confirmed with acute kidney injury, coronavirus disease 2019 (COVID-19), or undergoing cardiac surgery (Bayat et al., 2021; Tao et al., 2021; Fang et al., 2022; Gray et al., 2022; Xiong and Xiong, 2022; Zhou et al., 2022). Some studies have confirmed the pleiotropic effects of ondansetron, including anticoagulation, regulation of inflammation and immune status, renal protection, and neuroprotection (Fakhfouri et al., 2012; Liu et al., 2012; Sharma et al., 2019; Wu et al., 2019; Guo et al., 2021). One previous study showed that ondansetron treatment could attenuate blood–brain barrier breakdown, edema formation, decrease glial fibrillary acidic protein, and heat shock protein expression in rat models of morphine withdrawal (Sharma et al., 2019). In addition, one retrospective cohort study involving COVID-19 patients found that the long-term incidence of ischemic cerebral ischemia was lower in patients receiving ondansetron treatment (Bayat et al., 2021). The neuroprotective role of ondansetron has not been widely verified in other kinds of brain injury patients. Nausea and vomiting are common symptoms among TBI patients, with prevalence ranging from 25% to 30% (Feiz Disfani et al., 2022). Ondansetron is usually prescribed to effectively alleviate these symptoms of TBI patients. However, the effect of ondansetron on the prognosis of TBI patients has not been explored. Therefore, we conducted this study to verify the association between ondansetron treatment and mortality of TBI patients.

## 2 Materials and methods

### 2.1 Patients

**FIGURE 1:**
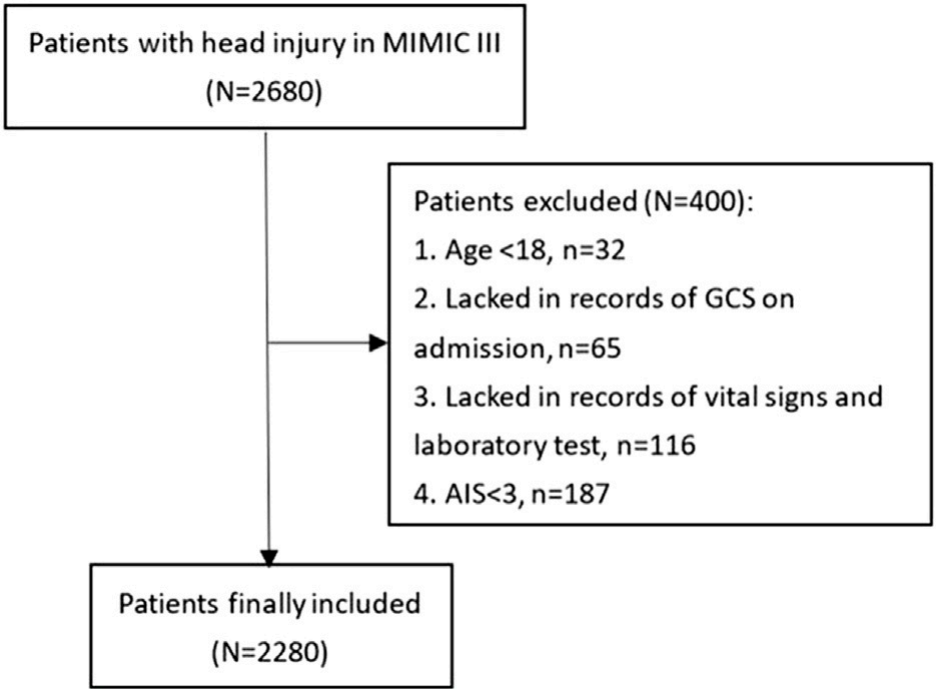
Flowchart of patients’ inclusion.

Patients were selected from the Medical Information Mart for Intensive Care-III (MIMIC-III) database, which is an intensive care database enrolling patients hospitalized in the Beth Israel Deaconess Medical Center (BIDMC) (Boston, MA) between 2001 and 2012. The MIMIC-III database was produced by the computational physiology laboratory of Massachusetts Institute of Technology (MIT) and obtained ethical approval from the review boards of MIT and BIDMC. All enrolling patients have been de-identified and anonymized to protect their privacy. We included 2,680 TBI patients for the study from the MIMIC-III database according to the following ICD-9 codes: 80000–80199, 80300–80499, and 8500–85419. Eligible patients were excluded according to following criteria: (1) age<18 (n = 32), (2) lacking records of the Glasgow Coma Scale (GCS) on admission (n = 65), (3) lacking records of vital signs and laboratory tests (n = 116), and (4) Abbreviated Injury Score (AIS) < 3 (n = 187). A total of 2,280 patients were finally included for analyses (Figure 1).

### 2.2 Data collection

Age, gender, and vital signs on admission, including systolic blood pressure, diastolic blood pressure, heart rate, and saturation of pulse oxygen (SpO_2_), were recorded. GCS and the Injury Severity Score (ISS) were selected as markers of injury severity. Comorbidities were also recorded, including diabetes, hypertension, hyperlipidemia, coronary heart disease, history of myocardial infarction, cerebral vascular disease, chronic liver disease, chronic renal disease, and cancer. Intracranial injury types were collected, including epidural hematoma, subdural hematoma, subarachnoid hemorrhage, and intraparenchymal hemorrhage. Laboratory tests, including white blood cell, platelet, red blood cell, red cell distribution width, hemoglobin, glucose, blood urea nitrogen, serum creatinine, serum sodium, and serum potassium, were acquired by analyzing the first blood sample on the first day after admission. Coagulopathy was diagnosed based on the following criteria: activated prothrombin time >40 s or/and international normalized ratio >1.2 or/and platelet <120×10^9^/L (Alexiou et al., 2014; Dekker et al., 2016). Medical treatments during the first 24 h, including vasopressor use, red cell transfusion, and platelet transfusion, were included as variables. The usage of ondansetron, including intravenous injection and oral tablet, since admission to the BIDMC was identified. The primary outcome of this study was the 30-day mortality. The length of intensive care unit (ICU) stay and length of hospital stay were compared between the ondansetron and non-ondansetron groups. All the mentioned variables were extracted using the Structured Query Language from the MIMIC-III database.

### 2.3 Statistical analysis

The normality of the included variables was verified by the Kolmogorov–Smirnov test. The normally distributed variables were shown as mean ± standard deviation, and the non-normally distributed variables were shown as median (interquartile range). The differences between the two groups of normally distributed variables and non-normally distributed variables were analyzed by the Student’s t-test or Mann–Whitney U test, respectively. Categorical variables were presented as counts (percentage). The difference between two groups of categorical variables was analyzed by the chi-squared test or Fisher’s exact test. The association between the ondansetron use and mortality of TBI patients was verified by the univariate and subsequent multivariate logistic regression. Then, propensity score matching (PSM) was conducted to generate baseline balanced cohorts (ondansetron and non-ondansetron groups) with a matching ratio of 1:1. The association between the ondansetron use and mortality of TBI patients was verified in the cohort after PSM by the logistic regression and Cox regression again. Sub-group analysis was also performed in the cohort after PSM with different TBI severity levels (GCS ≤12 and GCS >12).

The two-sided *p*-value < 0.05 was defined as statistically significant. R (version 3.6.1; R Foundation) was utilized to perform statistical analyses and draw figures.

## 3 Results

### 3.1 Baseline characteristics of TBI patients

In TBI cohorts before PSM, the usage incidence of ondansetron was 37.2% (847/2,280) (Table 1). Compared with the non-ondansetron group, the ondansetron group had lower age (*p* = 0.031), higher GCS (*p* < 0.001), and lower ISS (*p* < 0.001). Epidural hematoma (*p* = 0.006) and subdural hematoma (*p* = 0.001) were more frequently observed in the ondansetron group. In addition, the white blood cell (*p* = 0.001), red cell distribution width (*p* = 0.025), glucose (*p* = 0.001), and serum creatinine (*p* = 0.023) were lower in the ondansetron group, while platelet (*p* < 0.001), red blood cell (*p* = 0.001), and hemoglobin (*p* = 0.015) were higher in the ondansetron group. The ondansetron group was less likely to receive vasopressor (*p* < 0.001), red cell transfusion (*p* = 0.003), and mechanical ventilation (*p* < 0.001). The 30-day mortality was significantly lower in the ondansetron group (9.7% vs. 22.5%, *p* < 0.001). In TBI cohorts after PSM, the ondansetron group had a longer length of hospital stay (*p* < 0.001). The mortality of the ondansetron group was lower than that of the non-ondansetron group, although without statistical significance (10.2% vs. 13.1%, *p* = 0.079).

**TABLE 1:** Baseline characteristics of TBI patients divided by ondansetron use.

	Cohorts before PSM				Cohorts after PSM			
Variable	Overall patients (N = 2,280)	Non-ondansetron group (N = 1,433, 62.8%)	Ondansetron group (N = 847, 37.2%)	*p*	Overall patients (N = 1,536)	Non-ondansetron group (N = 768)	Ondansetron group (N = 768)	*p*
Age (year)	64.9 (43.7–81.0)	66.4 (44.3–81.6)	61.8 (42.6–80.0)	**0.031**	64.6 (43.2–81.1)	64.6 (41.8–81.4)	64.6 (44.5–81.0)	0.778
Male gender (%)	1,400 (61.4%)	921 (64.3%)	479 (56.6%)	**<0.001**	879 (57.7%)	441 (57.9%)	438 (57.5%)	0.876
Vital signs on admission								
Systolic blood pressure (mmHg)	132 (117–147)	132 (116–147)	132 (117–147)	0.870	133 (118–147)	133 (118–147)	133 (117–147)	0.759
Diastolic blood pressure (mmHg)	67 (56–77)	67 (56–77)	67 (56–78)	0.465	67 (57–77)	67 (57–77)	67 (56–78)	0.821
Heart rate (s^-1^)	83 (72–96)	84 (72–97)	82 (72–94)	**0.046**	82 (72–95)	82 (71–95)	82 (73–95)	0.998
SpO_2_ (%)	99 (97–100)	99 (97–100)	99 (97–100)	**0.006**	99 (97–100)	99 (97–100)	99 (97–100)	0.878
GCS	12 (6–15)	10 (6–15)	14 (9–15)	**<0.001**	14 (8–15)	14 (8–15)	14 (8–15)	0.314
ISS	16 (16–25)	16 (16–25)	16 (16–20)	**<0.001**	16 (16–21)	16 (16–21)	16 (16–21)	0.825
Comorbidities								
Diabetes (%)	351 (15.4%)	229 (16.0%)	122 (14.4%)	0.343	237 (15.6%)	120 (15.7%)	117 (15.4%)	0.832
Hypertension (%)	844 (37.0%)	537 (37.5%)	307 (36.2%)	0.588	572 (37.3%)	283 (37.1%)	289 (37.9%)	0.751
Hyperlipidemia (%)	298 (13.1%)	175 (12.2%)	123 (14.5%)	0.129	221 (14.5%)	110 (14.4%)	111 (14.6%)	0.942
Coronary heart disease (%)	293 (12.9%)	183 (12.8%)	110 (13.0%)	0.933	202 (13.3%)	99 (13.0%)	103 (13.5%)	0.763
History of myocardial infarction	83 (3.6%)	56 (3.9%)	27 (3.2%)	0.440	46 (3.0%)	20 (2.6%)	26 (3.4%)	0.369
Cerebral vascular disease (%)	41 (1.8%)	26 (1.8%)	15 (1.8%)	1.000	29 (1.9%)	15 (2.0%)	14 (1.8%)	0.851
Chronic liver disease	94 (4.1%)	64 (4.5%)	30 (3.5%)	0.335	62 (4.1%)	32 (4.2%)	30 (3.9%)	0.795
Chronic renal disease (%)	153 (6.7%)	97 (6.8%)	56 (6.6%)	0.953	110 (7.2%)	56 (7.3%)	54 (7.1%)	0.843
Cancer (%)	238 (10.4%)	137 (9.6%)	101 (11.9%)	0.087	175 (11.5%)	83 (10.9%)	92 (12.1%)	0.47
Intracranial injury type								
Epidural hematoma (%)	543 (23.8%)	314 (21.9%)	229 (27.0%)	**0.006**	386 (25.3%)	192 (25.2%)	194 (25.5%)	0.906
Subdural hematoma (%)	1,319 (57.9%)	789 (55.1%)	530 (62.6%)	**0.001**	930 (61.0%)	464 (60.9%)	466 (61.2%)	0.916
Subarachnoid hemorrhage (%)	958 (42.0%)	613 (42.8%)	345 (40.7%)	0.362	617 (40.5%)	305 (40.0%)	312 (40.9%)	0.715
Intraparenchymal hemorrhage (%)	447 (19.6%)	294 (20.5%)	153 (18.1%)	0.170	288 (18.9%)	143 (18.8%)	145 (19.0%)	0.896
Laboratory tests								
White blood cell (10^9/L)	11.60 (8.40–15.70)	11.80 (8.70–16.10)	11.30 (8.20–15.00)	**0.001**	11.40 (8.20–15.20)	11.40 (8.20–15.50)	11.40 (8.20–15.10)	0.802
Platelet (10^9/L)	230 (183–285)	225 (177–280)	241 (191–293)	**<0.001**	234 (189–288)	231 (189–288)	235 (188–289)	0.375
Red blood cell (10^9/L)	4.13 (3.67–4.57)	4.10 (3.63–4.53)	4.20 (3.74–4.62)	**0.001**	4.14 (3.71–4.59)	4.13 (3.71–4.58)	4.15 (3.71–4.60)	0.772
Red cell distribution width	13.5 (12.9–14.4)	13.6 (13.0–14.4)	13.4 (12.8–14.3)	**0.025**	13.5 (12.9–14.4)	13.5 (12.9–14.4)	13.5 (12.9–14.4)	0.891
Hemoglobin (g/dL)	12.8 (11.4–14.1)	12.7 (11.2–14.0)	12.9 (11.6–14.2)	**0.015**	12.80 (11.50–14.20)	12.80 (11.40–14.10)	12.80 (11.50–14.20)	0.815
Glucose (mg/dL)	132 (110–165)	134 (110–170)	128 (108–156)	**0.001**	128 (108–158)	124 (106–157)	131 (110–159)	0.073
Blood urea nitrogen (mg/dL)	16 (12–23)	17 (12–23)	16 (12–22)	0.157	16 (12–22)	16 (12–22)	16 (12–22)	0.567
Serum creatinine (mg/dL)	0.9 (0.7–1.1)	0.9 (0.8–1.1)	0.9 (0.7–1.1)	**0.023**	0.9 (0.7–1.1)	0.9 (0.7–1.1)	0.9 (0.7–1.1)	0.864
Serum sodium (mmol/L)	139 (137–141)	139 (137–142)	139 (137–141)	0.115	139 (137–141)	139 (137–141)	139 (137–141)	0.854
Serum potassium (mmol/L)	4.0 (3.7–4.4)	4.0 (3.7–4.4)	4.0 (3.7–4.4)	0.764	4 (3.7–4.4)	4.0 (3.7–4.3)	4.0 (3.7–4.4)	0.651
Coagulopathy	743 (32.6%)	512 (35.7%)	231 (27.3%)	**<0.001**	451 (29.6%)	229 (30.1%)	222 (29.1%)	0.694
Vasopressor during the first 24 h (%)	150 (6.6%)	126 (8.8%)	24 (2.8%)	**<0.001**	51 (3.3%)	27 (3.5%)	24 (3.2%)	0.669
Red cell transfusion during the first 24 h (%)	178 (7.8%)	131 (9.1%)	47 (5.5%)	**0.003**	86 (5.6%)	40 (5.2%)	46 (6.0%)	0.505
Platelet transfusion during first 24 h (%)	223 (9.8%)	139 (9.7%)	84 (9.9%)	0.924	147 (9.6%)	74 (9.7%)	73 (9.6%)	0.931
Mechanical ventilation (%)	1,034 (45.4%)	760 (53.0%)	274 (32.3%)	**<0.001**	535 (35.1%)	270 (35.4%)	265 (34.8%)	0.788
Neurosurgery (%)	572 (25.1%)	344 (24.0%)	228 (26.9%)	0.134	367 (24.1%)	174 (22.8%)	193 (25.3%)	0.255
Thirty-day mortality (%)	404 (17.7%)	322 (22.5%)	82 (9.7%)	**<0.001**	178 (11.7%)	100 (13.1%)	78 (10.2%)	0.079
Length of ICU stay (day)	2 (1–6)	3 (1–7)	2 (1–4)	**<0.001**	2 (1–4)	2 (1–4)	2 (1–5)	0.425
Length of hospital stay (day)	6 (4–12)	6 (3–13)	6 (4–12)	0.846	6 (4–12)	6 (3–11)	6 (4–12)	<0.001

SpO_2_, pulse oxygen saturation; GCS, Glasgow Coma Scale; ISS, Injury Severity Score.

Bold values mean *p* <0.05.

### 3.2 Effect of ondansetron on mortality of TBI patients

**FIGURE 2:**
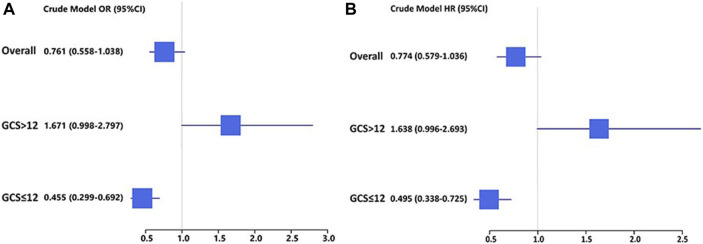
**(A)** Logistic regression analysis of the association between ondansetron use and mortality in mild (GCS > 12) and moderate-to-severe (GCS ≤ 12) TBI patients in cohorts after PSM; **(B)** Cox regression analysis of the association between ondansetron use and mortality in mild (GCS > 12) and moderate-to-severe (GCS ≤ 12) TBI patients in cohorts after PSM.

In TBI cohorts before PSM, univariate logistic regression showed that age (*p* < 0.001), diastolic blood pressure (*p* = 0.002), GCS (*p* < 0.001), ISS (*p* < 0.001), diabetes (*p* = 0.003), chronic renal disease (*p* < 0.001), cancer (*p* = 0.022), white blood cell (*p* < 0.001), platelet (*p* < 0.001), red blood cell (*p* < 0.001), red cell distribution width (*p* < 0.001), hemoglobin (*p* < 0.001), glucose (*p* < 0.001), blood urea nitrogen (*p* < 0.001), serum creatinine (*p* < 0.001), coagulopathy (*p* < 0.001), vasopressor use (*p* < 0.001), red cell transfusion (*p* < 0.001), platelet transfusion (*p* < 0.001), ondansetron (*p* < 0.001), mechanical ventilation (*p* < 0.001), and neurosurgery (*p* = 0.001) were associated with the mortality of TBI patients (Table 2). However, after adjusting the confounding effects, multivariate logistic regression showed 10 independent risk factors of mortality, including age (*p* < 0.001), GCS (*p* < 0.001), ISS (*p* < 0.001), chronic renal disease (*p* = 0.004), white blood cell (*p* = 0.004), red cell distribution width (*p* < 0.001), glucose (*p* = 0.002), coagulopathy (*p* = 0.018), ondansetron (*p* = 0.008), and mechanical ventilation (*p* < 0.001). In TBI cohorts after PSM, logistic regression indicated that ondansetron use was significantly associated with the lower mortality of moderate-to-severe TBI (*p* < 0.001) but not mild TBI (*p* = 0.051) (Table 3) (Figure 2). In addition, Cox regression also presented that ondansetron use was significantly associated with the lower mortality of moderate-to-severe TBI (*p* < 0.001) but not mild TBI (*p* = 0.052).

**TABLE 2:** Risk factors of mortality analyzed by univariate and multivariate logistic regression before PSM.

	Univariate logistic regression analysis			Multivariate logistic regression analysis		
Variables	OR	95% CI	*p*	OR	95% CI	*p*
Age	1.025	1.020–1.031	**<0.001**	1.049	1.040–1.058	**<0.001**
Male gender	0.881	0.708–1.097	0.257			
Systolic blood pressure	0.997	0.992–1.001	0.139			
Diastolic blood pressure	0.989	0.983–0.996	**0.002**	1.001	0.994–1.009	0.724
Heart rate	0.998	0.992–1.004	0.434			
SpO_2_	0.995	0.976–1.015	0.652			
GCS	0.819	0.798–0.841	**<0.001**	0.832	0.799–0.867	**<0.001**
ISS	1.047	1.036–1.059	**<0.001**	1.045	1.029–1.062	**<0.001**
Diabetes	1.521	1.156–2.002	**0.003**	0.875	0.603–1.269	0.482
Hypertension	1.128	0.905–1.407	0.283			
Hyperlipidemia	1.059	0.773–1.451	0.721			
Coronary heart disease	1.171	0.859–1.596	0.319			
History of myocardial infarction	0.858	0.470–1.566	0.617			
Cerebral vascular disease	1.313	0.622–2.772	0.475			
Chronic liver disease	0.951	0.549–1.646	0.856			
Chronic renal disease	2.431	1.702–3.473	**<0.001**	2.145	1.277–3.603	**0.004**
Cancer	1.458	1.056–2.013	**0.022**	1.173	0.776–1.773	0.449
Epidural hematoma	1.064	0.829–1.366	0.626			
Subdural hematoma	1.003	0.807–1.248	0.975			
Subarachnoid hemorrhage	1.176	0.947–1.461	0.141			
Intraparenchymal hemorrhage	0.833	0.629–1.104	0.204			
White blood cell	1.039	1.023–1.055	**<0.001**	1.032	1.010–1.055	**0.004**
Platelet	0.997	0.996–0.999	**<0.001**	0.999	0.997–1.000	0.091
Red blood cell	0.572	0.491–0.668	**<0.001**	0.995	0.652–1.519	0.981
Red cell distribution width	1.214	1.146–1.287	**<0.001**	1.171	1.074–1.277	**<0.001**
Hemoglobin	0.816	0.776–0.859	**<0.001**	0.992	0.855–1.150	0.911
Glucose	1.008	1.007–1.010	**<0.001**	1.003	1.001–1.006	**0.002**
Blood urea nitrogen	1.024	1.016–1.032	**<0.001**	1.002	0.989–1.015	0.793
Serum creatinine	1.260	1.116–1.422	**<0.001**	0.978	0.787–1.214	0.840
Serum sodium	1.020	0.994–1.046	0.135			
Serum potassium	0.972	0.831–1.137	0.721			
Coagulopathy	4.295	3.050–6.048	**<0.001**	1.673	1.091–2.564	**0.018**
Vasopressor during the first 24 h	2.674	1.921–3.721	**<0.001**	1.184	0.767–1.828	0.446
Red cell transfusion during the first 24 h	2.247	1.651–3.058	**<0.001**	0.990	0.657–1.492	0.963
Platelet transfusion during the first 24 h	2.407	1.933–2.996	**<0.001**	1.256	0.942–1.675	0.121
Ondansetron	0.370	0.285–0.479	**<0.001**	0.661	0.485–0.900	**0.008**
Mechanical ventilation	4.627	3.629–5.899	**<0.001**	1.924	1.353–2.736	**<0.001**
Neurosurgery	1.474	1.165–1.866	**0.001**	1.103	0.816–1.490	0.525

OR, odds ratio; CI, confidence interval; SpO_2_; pulse oxygen saturation; GCS, Glasgow Coma Scale; ISS, Injury Severity Score.

Bold values mean *p* <0.05.

**TABLE 3:** Association between ondansetron use and mortality in TBI sub-groups after PSM analyzed by the logistic regression and Cox regression.

	OR	95% CI	*p*	HR	95% CI	*p*
Overall patients	0.761	0.558–1.038	0.085	0.774	0.579–1.036	0.085
Mild TBI	1.671	0.998–2.797	0.051	1.638	0.996–2.693	0.052
Moderate-to-severe TBI	0.455	0.299–0.692	**<0.001**	0.495	0.338–0.725	**<0.001**

OR, odds ratio; CI, confidence interval; HR, hazard ratio.

Bold values mean *p* <0.05.

## 4 Discussion

Ondansetron is a widely used antiemetic drug for patients receiving radio-chemotherapy or undergoing surgery. In our study, ondansetron was commonly used among TBI patients, with the incidence of 37.2%. The ondansetron group of TBI patients in our study had higher GCS than the non-ondansetron group, which indicated that TBI patients with a better status of consciousness may be more likely to present nausea and vomiting. Furthermore, the sub-group analysis of our study indicated that ondansetron treatment was significantly associated with the mortality of moderate-to-severe TBI patients but not mild TBI patients. The effect of ondansetron on the mortality outcome has also been verified among other kinds of critically ill patients, including those diagnosed with acute kidney injury, COVID-19, or undergoing cardiac surgery (Bayat et al., 2021; Tao et al., 2021; Fang et al., 2022; Gray et al., 2022; Xiong and Xiong, 2022; Zhou et al., 2022). This beneficial influence may not only depend on the antiemetic effect but also on other effects of ondansetron, including anticoagulation, regulation of inflammation and immune status, renal protection, and neuroprotection (Fakhfouri et al., 2012; Liu et al., 2012; Sharma et al., 2019; Wu et al., 2019; Guo et al., 2021).

One animal study of morphine withdrawal showed that blood–brain barrier breakdown, edema formation, and the production of glial fibrillary acidic protein and heat shock protein could be attenuated by ondansetron treatment (Sharma et al., 2019). Additionally, ondansetron could inhibit the platelet aggregation by reducing agonist-induced inositol 1,4,5-triphosphate production and mitogen-activated protein kinases phosphorylation, which results in decreased intracellular Ca^2+^ mobilization, thromboxane B2 formation, and adenosine triphosphate release (Liu et al., 2012). A retrospective cohort study showed that the effect of ondansetron on lowering the rates of venous thromboembolisms among hospitalized patients was similar to that of aspirin (Datta et al., 2021). Furthermore, some studies reported the anti-inflammatory role of ondansetron in animal models of pancreatitis, colitis, and hepatic injury (Liu et al., 2011; Motavallian-Naeini et al., 2012; Tsukamoto et al., 2017). Two other studies found that 5-HT receptor antagonists, including sarpogrelate and tropisetron, could decrease the production of pro-inflammatory cytokines in shock models (Nishiyama, 2009; Setoguchi et al., 2011). In addition, ondansetron has been used to control symptoms of neuropsychiatric diseases, including obsessive compulsive disorder, through decreasing the dopaminergic activity and the release of serotonin, norepinephrine, and acetylcholine, which also play an important role in the pathophysiological process of TBI (Eissazade et al., 2023; Khan et al., 2023). Therefore, we reasonably hypothesize that ondansetron could improve the prognosis of TBI by regulating coagulation, inflammation, and immune status and protecting the kidney and brain after an injury. In addition, ondansetron may reduce the risk of pulmonary complications through decreasing vomiting-induced aspiration and excessive inflammation in the lungs.

Our observational study analyzes the relationship between ondansetron use and outcomes of TBI patients, which provides a new perspective and opportunity to develop therapeutic strategies for TBI. The causal relationship between ondansetron use and outcomes of TBI and the influence of ondansetron on pathophysiological changes of injured organs after TBI should be explored in future animal studies. Randomized controlled trials could be performed to verify the interventional effect of ondansetron use on the prognosis of moderate-to-severe TBI and further explore the optimal dose of ondansetron to improve the prognosis of moderate-to-severe TBI. Additionally, although ondansetron use was not found to be related to the mortality risk of mild TBI patients, it is still worth being prescribed in the conventional dose for mild TBI to alleviate nausea and vomiting.

This study has several limitations. First, confounding factors may not be totally adjusted due to the nature of an observational study. Future prospective randomized trials should be performed to verify the effect of ondansetron on the prognosis of TBI. Second, the primary outcome of this study was the 30-day mortality. Other outcomes, including functional status and cognitive status, were not recorded in the database so that we could not analyze the relationship between ondansetron and these outcomes. Third, we did not specifically analyze the administration time and dosage of ondansetron, although recent studies recommended a daily dose of ondansetron of no more than 16 mg (Fang et al., 2022; Sutherland et al., 2022). Fourth, we did not explore the effect of ondansetron on QT interval prolongation, although one previous study confirmed no significant changes of electrocardiogram parameters including QT interval after ondansetron administration (Assaad et al., 2023). Finally, we did not include other 5-HT3 receptor antagonists such as granisetron or tropisetron in the analysis due to their rare usage in the MIMIC-III database.

## 5 Conclusion

Considering the prognostic effect, ondansetron administration is associated with the improved survival outcome of moderate-to-severe TBI but not mild TBI patients. The clinical effect, optimal dose, and timing of ondansetron use for improving the prognosis of moderate-to-severe TBI are worth exploring in future studies.

## Data Availability

The raw data supporting the conclusion of this article will be made available by the authors, without undue reservation.
